# Temporal Effects of Sleeve Gastrectomy on Glucose-Insulin Homeostasis and Incretin Hormone Response at 1 and 6 Months

**DOI:** 10.1007/s11695-020-04457-9

**Published:** 2020-02-17

**Authors:** S. L. Prior, R. Churm, T. Min, G. J. Dunseath, J. D. Barry, J. W. Stephens

**Affiliations:** 1 Diabetes Research Group, Institute of Life Sciences, Swansea University, Swansea, SA2 8PP UK; 2 Applied Sports, Technology, Exercise and Medicine (A-STEM) Research Centre, Swansea University, Swansea, UK; 3 Department of Diabetes & Endocrinology, Morriston Hospital, Swansea Bay University Health Board, Swansea, UK; 4 Welsh Institute of Metabolic and Obesity Surgery, Morriston Hospital, Swansea Bay University Health Board, Swansea, UK

**Keywords:** Type 2 diabetes, Impaired glucose tolerance, Incretin, Sleeve gastrectomy

## Abstract

**Background:**

Bariatric surgery is an effective treatment for morbid obesity and glycaemic dysfunction.

**Objectives:**

The aim of the work was to examine both the static and dynamic changes of glucose-insulin homeostasis and incretin hormone response following sleeve gastrectomy (SG) in a sample of 55 participants preoperatively and 1 month and 6 months postoperatively. The focus was on a sample of patients with impaired glucose tolerance and type 2 diabetes (T2D).

**Setting:**

Morriston Hospital, UK.

**Methods:**

Prospective study comprising of 55 participants with impaired glucose homeostasis and T2D undergoing SG (mean body mass index [BMI] 50.4 kg/m^2^, mean glycated haemoglobin [A1C] 7.4%). Serial measurements of glucose, insulin, C-peptide, glucagon-like peptide-1 (GLP-1) and glucose-dependent insulinotropic hormone (GIP) were performed during oral glucose tolerance testing preoperatively and 1 and 6 months postoperatively. Areas under the curve (AUC) were examined at 30, 60, and 120 min.

**Results:**

We observed significant improvements in measures of obesity, as well as static and dynamic measures of glucose, insulin, C-peptide and HOMA. Furthermore, significant increases in GLP-1 response as early as 6 months postoperatively were also seen.

**Conclusions:**

To our knowledge, no study has examined the detailed dynamic changes in glucose and insulin homeostasis in this number of participants undergoing SG in relation to incretin hormones GIP and GLP-1. This current study supports the role of SG for the treatment of obesity-related glucose dysregulation.

## Introduction

Obesity is associated with a number of co-morbidities including diabetes, cardiovascular disease, hypertension and osteoarthritis [1]. Increased rates of obesity contribute a substantial financial burden, especially in the USA, where obesity is associated with an additional annual medical expenditure of $1900 per person. This equates to a national excess healthcare expenditure of $150 billion for people with obesity [2]. Bariatric surgery is an effective treatment for morbid obesity and is associated with at least 80% remission of impaired glucose tolerance (IGT) and type 2 diabetes (T2D) [3–7]. Bariatric procedures were traditionally described as being restrictive, malabsorptive or a combination of both. Sleeve gastrectomy (SG) has attracted considerable surgical interest as it does not require intestinal bypass or anastomosis and is considered less technically challenging than other malabsorptive procedures. Although SG is anatomically a restrictive procedure, its mechanism of action is more complex. The SG procedure requires the removal of gastric cells that produce orexigenic hormones and may be associated with changes in incretin hormones. These anatomical and physiological changes may explain its superiority over other restrictive procedures in the management of excess weight and impaired glucose regulation [8, 9].

Incretin hormones are associated with the metabolic outcome of bariatric surgery [10, 11]. Of note, the incretin hormones contribute between 50 and 70% of the total postprandial insulin release, mainly through the actions of glucagon-like peptide-1 (GLP-1) and glucose-dependent insulinotropic polypeptide (GIP) [12, 13]. SG, which involves removal of the gastric fundus and body, results in an increased rate of gastric emptying and subsequently more rapid contact of postprandial nutrients with the apical surface of the intestinal L cells resulting in stimulation of GLP-1 release [14]. We have previously published the findings of a small study examining the postprandial temporal relationship of markers of glucose, insulin and gut hormone homeostasis in 22 participants undergoing SG [15]. The study demonstrated significant early improvements in insulin sensitivity and incretin hormone response, along with improvements in IGT/T2D [15]. Subsequently, other studies have reported on the association between incretin hormones, glucose and insulin homeostasis; however, patient numbers are small, and much of the focus has been on other surgical techniques such as Roux-en-Y gastric bypass (RYGB) [10, 16]. The specific aims of the current study were to examine changes in glucose-insulin homeostasis and incretin hormone response at 1 and 6 months following SG in an extended sample of 55 participants.

## Methods

### Recruitment

Approval for the study was obtained from the local research ethics committee. Participants were identified and recruited from those undergoing a planned bariatric surgical procedure. This study was an extension of a previous study where 22 participants were recruited. The background details have been previously been published [15]. Entry criteria at the outset included the following: both genders, age 20–60 years, body mass index (BMI) > 40 kg/m^2^ and physically fit for surgery. Participants with pre-existing T2D treated with diet, oral agents, GLP-1 analogues or insulin were included. Participants with impaired glucose regulation were those with either impaired fasting glycaemia (5.6–6.9 mmol/L) or impaired glucose tolerance (2-h glucose value between 7.8 and 11.0 mmol/L) were also included. All participants were recruited within 1 month preoperatively and followed up postoperatively at 1 and 6 months where they underwent a standardized 75-g oral glucose tolerance test (OGTT) (122-mL Polycal 61.9 g/100-mL glucose, Nutricia Clinical Care, Trowbridge, UK). All diabetes-related agents were omitted for 24 h before the OGTT. There was no standardized meal prescribed for the night before, and participants were asked to fast from the midnight before the test. All participants with the help of the research nurse completed a preoperative questionnaire, and all clinical measurements were documented during the visits. A total of 55 participants completed the extension study.

### Preoperative Clinical and Biochemical Information

As described previously [15], preoperative clinical measurements consisted of weight, height, BMI, waist circumference and systolic and diastolic blood pressure. Preoperative biochemical measurements (total cholesterol, low-density lipoprotein cholesterol [LDL-C], high-density lipoprotein cholesterol [HDL-C] and triglycerides) were analysed within the local hospital accredited laboratory. During the OGTT, plasma and serum samples were collected for measurements of glucose, insulin, C-peptide, GLP-1 and GIP at time 0, 15, 30, 45, 60 and 120 min. All samples were collected on ice, centrifuged and separated within 1 h of collection and subsequently stored at − 80 °C until analysis.

### Measurement of Glucose, Insulin, C-Peptide and Estimation/Calculation of Insulin Sensitivity and β Cell Function

Glucose was measured using a Randox Daytona-plus Clinical Chemistry analyser, via a colorimetric glucose oxidase method, with an analytical sensitivity of 0.02 mmol/L and a dynamic range of 0.02-250 mmol/L. The inter-assay coefficient of variation was ≤ 7.1%.

Insulin was measured using an Invitron Insulin ELISA kit, with an analytical sensitivity of 0.02 mU/L and a dynamic range of 0.02–250 mU/L. The inter-assay coefficient of variation was ≤ 7.1%. No high-dose hook effect was observed at insulin concentrations up to 20,000 mU/L. Cross-reactivities (CR) of related proteins were as follows: 1.2% with intact proinsulin and 0% with C-peptide.

C-peptide was measured with an Invitron C-peptide kit, with an analytical sensitivity of 5.0 pmol/L and a dynamic range of 5.0–5000 pmol/L. No high-dose hook effect was observed at C-peptide concentrations up to 30,000 pmol/L. There is 2% cross-reactivity with intact proinsulin but no-cross-reactivity with insulin.

Insulin sensitivity and β cell function were calculated using the HOMA-2 calculator, utilizing measurements of fasting glucose and insulin concentrations. These were calculated by using the Oxford University online calculator (https://www.dtu.ox.ac.uk/homacalculator/; accessed 01 June 2015). HOMA provides three measures: HOMA-%B (estimated steady state β cell function), HOMA-%S (insulin sensitivity) and HOMA-IR (insulin resistance). These measures have been validated and shown to correlate with clamp-derived studies [17].

For calculating early-phase (ΔI_0–30_/ΔG_0–30_) and late-phase (ΔI_60–120_/ΔG_60–120_) insulin response, published formulas using insulin and glucose values obtained during OGTT time points were used [18, 19].

### Measurement of Total GLP-1 and Total GIP

Total GLP-1 was quantitatively measured using the EMD Millipore Total GLP-1 ELISA Kit. The antibody pair used in this assay measures GLP-1 (7–36) and (9–36) and has no significant cross-reactivity with GLP-2, GIP, glucagon or oxyntomodulin. The sensitivity of this assay is 1.5 pmol/L, and the approximate range is 4.1–1000 pmol/L. The intra- and inter-assay coefficients of variation were ≤ 2% and ≤ 12%, respectively. Total GIP was measured using the EMD Millipore Human GIP (total) ELISA Kit, which reacts fully with intact GIP (1–42) and the NH_2_-terminally truncated metabolite GIP (3–42). The assay does not significantly cross-react with glucagon, oxyntomodulin, GLP-1 or GLP-2. The sensitivity of this assay is 4.2 pg/mL, with an assay range of 4.2–2000 pg/mL. The intra- and inter-assay coefficients of variation were ≤ 8.8% and ≤ 6.1%, respectively.

### Statistical Analysis

Statistical analysis was performed using Statistical Package for the Social Sciences (SPSS, Version 22). All results for continuous variables are presented as median and interquartile range (IQR) as none were normal distributed and in graphical representation as mean and standard error. Differences between preoperative and postoperative measurements at 1 and 6 months were compared using a Wilcoxon signed-rank test. Paired t tests were used to compare differences at individual time points during the OGTT between preoperative and 1 and 6 months. Area under the curve (AUC) over 30 min (AUC_0–30_), 60 min (AUC_0–60_) and 120 min (AUC_0–120_) were analysed during the OGTT at preoperative, and 1 and 6 months using the trapezoidal rule. In all cases, a *P* value below 0.05 was considered statistically significant.

## Results

### Participant Characteristics

A total of 55 participants (31 females and 24 males) who underwent SG completed the study, with a mean age of 46 ± 8 years. The preoperative characteristics along with the changes in anthropometric and clinical measures are summarized in Table 1. Significant reductions were observed at 1 and 6 months following SG in relation to measures of obesity and plasma triglyceride levels. There was also a significant increase in HDL cholesterol.

**Table 1:** Baseline preoperative and postoperative characteristics of the study sample

Measurement	Preoperative	1 month	*P* value^*^	6 months	*P* value^†^
Weight (kg)	146.4 [129–171]	129.8 [107–144]	**< 0.001**	114.2 [102–124]	**< 0.001**
BMI (kg/m^2^)	50.5 [45.0–54.0]	44.4 [38.0–49.2]	**< 0.001**	38.2 [34.1–41.9]	**< 0.001**
Waist (cm)	140 [127–152]	124 [114–142]	**< 0.001**	116 [107–128]	**< 0.001**
Systolic BP (mmHg)	126 [115–134]	120 [111–133]	**0.044**	120 [112–136]	0.117
Diastolic BP (mmHg)	74 [68–83]	75 [67–79]	0.097	75 [65–79]	0.053
Cholesterol (mmol/L)	4.3 [3.5–5.0]	4.0 [3.3–4.9]	0.820	4.3 [3.8–5.2]	0.124
LDL (mmol/L)	2.2 [1.8–2.9]	2.2 [1.7–3.1]	0.237	2.6 [2.0–3.3]	**0.027**
HDL (mmol/L)	1.1 [0.9–1.3]	1.0 [0.9–1.2]	**0.002**	1.2 [1.0–1.4]	**< 0.001**
Triglyceride (mmol/L)	1.5 [1.1–2.4]	1.4 [1.1–1.9]	0.102	1.2 [0.9–1.6]	**0.002**

Median and interquartile ranges shown

^*^P value comparing preoperative with 1 month

†*P* value comparing preoperative with 6 months

Significant values are in bold. BMI = body mass index. BP = blood pressure. LDL = low-density lipoproteins. HDL = high-density lipoproteins

### Static and Dynamic Changes in Glucose-Insulin Homeostasis Following SG

Tables 2 and 3 show the postoperative changes in the static and dynamic measures relating to glucose, insulin, C-peptide and HOMA. As seen in Table 2, at 1 and 6 months following SG, significant reductions were observed in glycaemic measurements (A1C, fasting and 2-h plasma glucose). Of particular note, A1C had improved markedly by 6 months falling below the diagnostic cut-off. Consistent with a reduction in insulin resistance, there were also significant improvements in fasting insulin and fasting C-peptide levels. In line with these observations, there were significant improvements of approximately 50% in markers of insulin resistance (HOMA-IR) and insulin sensitivity (HOMA-%S).

**Table 2:** Preoperative and postoperative static changes in glycaemic measures

Measurement	Preoperative	1 month	*P* value^*^	6 months	*P* value^†^
A1C (mmol/mol)	57.0 [46.0–89.3]	46.0 [38.5–56.0]	**< 0.001**	40.0 [36.0–49.0]	**< 0.001**
A1C (%)	7.4 [6.4–10.3]	6.3 [5.6–7.3]	**< 0.001**	5.8 [5.4–6.6]	**< 0.001**
Fasting glucose (mmol/L)	7.1 [5.9–11.7]	5.6 [4.6–6.8]	**< 0.001**	5.2 [4.5–5.8]	**< 0.001**
2-h glucose (mmol/L)	13.4 [9.2–18.4]	8.8 [5.2–12.7]	**< 0.001**	5.8 [4.2–9.4]	**< 0.001**
Fasting insulin (mU/L)	21.8 [13.7–29.5]	12.0 [8.9–19.3]	**< 0.001**	9.0 [5.4–14.1]	**< 0.001**
2-h insulin (mU/L)	52.8 [27.4–102.4]	47.1 [27.7–122.6]	0.210	29.7 [16.2–56.9]	**0.010**
Fasting C-peptide (pmol/mL)	4.0 [3.5–5.2]	3.6 [2.7–4.8]	**0.002**	2.8 [2.0–3.9]	**< 0.001**
2-h C-peptide (pmol/mL)	8.7 [6.5–11.2]	10.0 [7.7–12.5]	0.058	8.7 [5.9–11.5]	0.868
HOMA-IR	3.1 [1.9–4.1]	1.6 [1.2–2.6]	**< 0.001**	1.3 [0.8–1.9]	**< 0.001**
HOMA-%B	90.1 [36.2–131.6]	110.0 [73.0–147.0]	0.343	99.5 [81.0–150.2]	0.288
HOMA-%S	32.4 [24.5–52.3]	62.3 [39.0–81.7]	**< 0.001**	75.5 [52.3–121.0]	**< 0.001**

Median and interquartile ranges shown

^*^P value comparing preoperative with 1 month

^†^*P* value comparing preoperative with 6 months

Significant values are in bold. A1C = glycated haemoglobin. HOMA-IR = homeostatic model assessment insulin resistance. HOMA-%S = homeostatic model assessment insulin sensitivity. HOMA-%B = homeostatic model assessment β cell function

**Table 3:** Preoperative and postoperative dynamic changes in glycaemic measures

	Preoperative	1 month	*P* value^*^	6 months	*P* value^†^
Early Phase (ΔI_0–30_/ΔG_0–30_)	5.3 [1.8–13.0]	10.0 [5.9–16.3]	0.172	11.9 [5.9–17.4]	**< 0.01**
Late Phase (ΔI_60–120_/ΔG_60–120_)	3.6 [−0.5–12.4]	13.5 [5.0–21.3]	0.157	11.6 [4.8–21.3]	**< 0.01**
**AUC**_**0–30**_					
Glucose (mmol h L^−1^)	4.6 [3.7–7.0]	4.2 [3.6–5.1]	0.154	4.0 [3.4–4.4]	**< 0.001**
Insulin (mU h L^−1^)	16.6 [10.0–25.4]	23.0 [13.7–33.7]	**0.002**	17.5 [14.4–30.1]	**0.005**
C-peptide (pmol h mL^−1^)	2.5 [1.9–3.1]	3.0 [2.5–3.7]	**< 0.001**	2.4 [2.0–3.4]	**0.020**
**AUC**_**0–60**_					
Glucose (mmol h L^−1^)	11.3 [9.1–17.0]	10.2 [8.7–12.6]	0.066	9.0 [7.7–11.8]	**< 0.001**
Insulin (mU h L^−1^)	46.9 [27.8–74.3]	69.8 [42.8–92.5]	**0.001**	61.0 [37.6–90.7]	**0.005**
C-peptide (pmol h mL^−1^)	6.1 [4.6–7.8]	7.5 [6.6–9.6]	**< 0.001**	6.5 [5.3–9.0]	**< 0.001**
**AUC**_**0–120**_					
Glucose (mmol h L^−1^)	24.3 [19.1–36.4]	19.8 [17.3–25.7]	**0.002**	17.3 [14.6–24.9]	**< 0.001**
Insulin (mU h L^−1^)	99.6 [60.1–197.8]	154.9 [99.8–202.9]	**0.029**	117.6 [75.6–204.9]	0.129
C-peptide (pmol h mL^−1^)	13.9 [11.1–18.8]	18.5 [15.5–22.9]	**< 0.001**	15.9 [12.2–20.6]	**0.001**

Median and interquartile ranges shown

^*^P value comparing preoperative with 1 month

^†^*P* value comparing preoperative with 6 months

Significant values are in bold. AUC = area under the curve

**Fig. 1:**
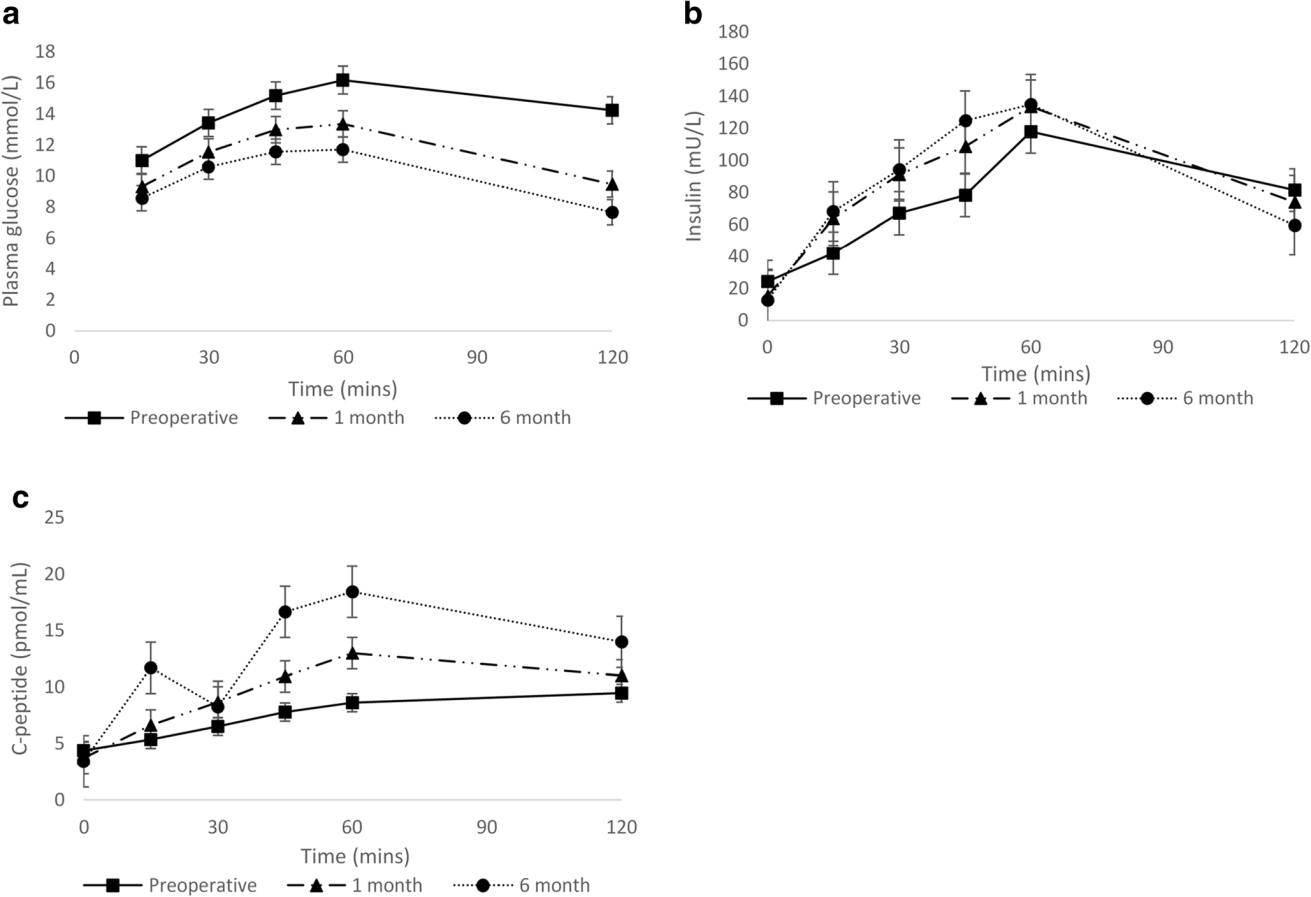
Changes in **a** glucose, **b** insulin and **c** C-peptide, during the 2-h oral glucose tolerance test (OGTT). Mean and standard error are shown. Mins = minutes

Significant changes were observed in both the early-phase (ΔI_0–30_/ΔG_0–30_) and late-phase (ΔI_60–120_/ΔG_60–120_) insulin release at 1 and 6 months postoperatively (Table 3). Significant reductions were observed in the AUC for glucose and C-peptide (Fig. 1a and c) and an increase in the AUC for insulin. This effect was observed at both AUC_0–30_ and AUC_0–60_, consistent with an improved early-phase insulin release following SG at 1 month and 6 months (Fig. 1b).

### Static and Dynamic Changes in Incretin Hormone Response Following SG

**Fig. 2:**
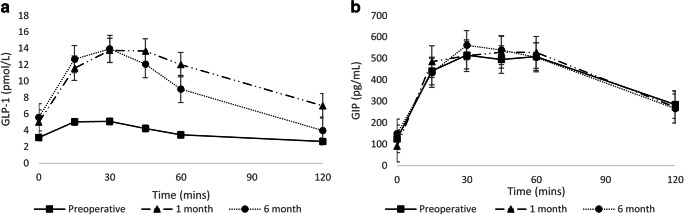
Changes in **a** glucagon-like peptide-1 (GLP-1) and **b** glucose-dependent insulinotropic hormone (GIP) during the 2-h oral glucose tolerance test (OGTT). Mean and standard error are shown. Mins = minutes

As shown in Table 4, there were no significant differences in the static levels of both GLP-1 and GIP measured at any of the visits. As expected, no changes were observed in fasting GLP-1 as this is an incretin hormone, which changes in relation to a nutrient load. Of note, the static 2-h GLP-1 levels increased at 1 and 6 months (*P* values of 0.067 and 0.060 respectively). However, as shown in Table 4 and Fig. 2 a, the AUC (AUC_0–30_, AUC_0–60_ and AUC_0–120_) for GLP-1 was significantly greater at 1 and 6 months postoperatively. Of interest early changes in the AUC were observed for GIP, but these effects were not maintained at 6 months (Table 4, Fig. 2b).

**Table 4:** Preoperative and postoperative static and dynamic changes in incretin hormones

Measurement	Preoperative	1 month	*P* value^*^	6 months	*P* value^†^
Fasting GLP-1 (pmol/L)	1.77 [1.1–4.2]	1.70 [0.4–5.2]	0.354	1.13 [0.6–4.5]	0.195
2-h GLP-1 (pmol/L)	1.49 [0.8–3.8]	3.91 [1.3–6.7]	0.067	2.48 [1.3–7.0]	0.060
Fasting GIP (pg/mL)	69.2 [48.0–123.3]	60.8 [40.9–86.8]	0.583	58.8 [35.1–87.0]	0.555
2-h GIP (pg/mL)	215.8 [156.7–348.7]	196.1 [127.2–276.6]	0.990	206.0 [125.6–281.4]	0.377
**AUC**_**0–30**_					
GLP-1 (pmol h L^−1^)	1.5 [0.9–2.7]	4.2 [2.3–5.9]	**< 0.001**	3.6 [2.1–7.0]	**< 0.001**
GIP (pg h mL^−1^)	163.3 [105.5–245.3]	154.0 [126.2–250.5]	**0.013**	150.2 [118.8–237.5]	0.272
**AUC**_**0–60**_					
GLP-1 (pmol h L^−1^)	3.2 [1.9–5.9]	9.6 [5.3–13.9]	**< 0.001**	8.2 [4.2–13.6]	**< 0.001**
GIP (pg h mL^−1^)	363.8 [252.4–550.3]	362.6 [282.7–610.4]	**0.010**	363.1 [273.5–582.2]	0.214
**AUC**_**0–120**_					
GLP-1 (pmol h L^−1^)	5.1 [3.2–10.6]	14.8 [8.8–24.7]	**< 0.001**	13.5 [6.4–22.3]	**< 0.001**
GIP (pg h mL^−1^)	710.2 [499.5–1043.5]	685.5 [525.3–1092.1]	**0.049**	711.6 [508.3–998.0]	0.717

Median and interquartile ranges shown

^*^P value comparing preoperative with 1 month

^†^*P* value comparing preoperative with 6 months

Significant values are in bold. AUC = area under the curve. GLP-1 = glucagon-like peptide-1. GIP = glucose-dependent insulinotropic hormone

## Discussion

This study conducted in 55 participants demonstrates that SG is an effective procedure to improve glucose-insulin homeostasis in patients with impaired glucose regulation and T2D. Significant improvements were observed in measures of obesity, as well as static and dynamic measures of glucose, insulin, C-peptide and HOMA. Furthermore, significant increases in GLP-1 response as early as 6 months postoperatively were also seen.

Previously published studies include samples with a small number of participants and furthermore lack detailed analysis of dynamic measures of insulin-glucose homeostasis and incretin hormones conducted during an OGTT. This current study has examined these effects in 55 participants with detailed follow-up. In line with previous studies in smaller samples, we observed significant improvement in glucose homeostasis, with decreases in A1C, fasting and 2-h glucose [20–23]. In addition, we observed significant improvements in measures of insulin resistance and insulin sensitivity as measured by HOMA-IR and HOMA-%S at both 1 and 6 months postoperatively. This is also reflected in the dynamic measures for glucose-insulin homeostasis, with significant changes in the AUC analysis for glucose, insulin and C-peptide. Gill et al. examined the effect of SG on participants with T2D, reporting after a mean follow-up of 13.1 months, with 26.9% showing improvements in glycaemic control, and a mean reduction of −1.7% (−18.0 mmol/mol) in A1C [24, 25]. The beneficial effects of SG on glucose homeostasis were also reported in improvements of A1C across multiple studies 6 months postoperatively, with reductions in A1C by 2.5% [26] and 1.6% [27]. However, a comparable A1C reduction of 1.6% was seen in a cohort with T2D and a substantially lower baseline BMI of 27.7 kg/m^2^ [28], indicating diabetes remission is weight independent.

As expected, within this study, fasting GLP-1 levels showed no change postoperatively. This is not surprising as GLP-1 is an incretin hormone, with its release being nutrient dependent. A significant increase in the postprandial GLP-1 response was observed postoperatively [15], a finding supported by a recent meta-analysis by McCarty et al. looking at 11 studies totalling *n* = 168 participants [29]. Sista et al. report significant increase of 1.1 pg/mL in dynamic GLP-1 levels, 6 months postoperatively. Metabolic profiles also alter post Roux-en-Y gastric bypass (RYGB), with increased GLP-1 levels and an early exaggerated insulin response [30, 31]. Several studies have reported an association between gut hormone levels, in particular GLP-1, and improvements in insulin secretion. This relationship is shown to be independent of weight loss and neurohormonal changes seen to also be observed post-SG in association with early-phase insulin release [32, 33]. Of interest, we observed decreases in GIP levels (non-significant) postoperatively, which has previously been observed by McCarty et al. during their meta-analysis of two studies that totalling *n* = 30 participants that measured GIP hormone levels before and after SG [29]. Previous publications have suggested that a primary role for GIP in the postprandial state may be to stimulate insulin secretion [34]. We hypothesize that the glycaemic changes seen post-SG are due to both GIP and GLP-1 s incretin action to normalize blood glucose levels via increased insulin synthesis and improved peripheral insulin sensitivity. Of interest, a recent publication by Kim et al. suggested that even though there is a substantially increase in intestinally derived GLP-1 following SG, it is the pancreatic α cell-derived peptides that are necessary for the surgery-induced improvements in glucose homeostasis [35]. However, these studies were conducted in a murine model, so the paracrine action of GLP-1 could play a more important role in mice than in humans but could be an alternative avenue of investigation in human participants in the future.

## Limitations

There are limitations to the current study. First was the study design, which was a non-randomized prospective study and as such had no control group. Additionally, we did not plan at the outset to measure other gut hormones such as ghrelin or neuropeptide Y.

## Conclusion

To our knowledge, no study has examined the detailed dynamic changes in glucose and insulin homeostasis in this number of participants undergoing SG in relation to incretin hormones GIP and GLP-1. SG is becoming more popular as the operation of choice in the field of bariatric surgery and is associated with early postoperative discharge from hospital, lower rates of associated complications and nutrient deficiencies. It is now clear that the early improvement in glycaemic control is associated with early changes in GLP-1 physiology, improved insulin-glucose homeostasis and improvements in insulin sensitivity. The current study adds to the available evidence supporting SG as a stand-alone procedure for the management of obesity-related glucose dysregulation.
